# Gaps in the care cascade for screening and treatment of refugees with tuberculosis infection in Middle Tennessee: a retrospective cohort study

**DOI:** 10.1186/s12879-020-05311-0

**Published:** 2020-08-10

**Authors:** Adoma Manful, Leslie Waller, Ben Katz, Jason Cummins, Jon Warkentin, Billy Reagon, Joanna Shaw-Kaikai, Yuwei Zhu, Yuri F. van der Heijden

**Affiliations:** 1 Vanderbilt University School of Medicine, Nashville, USA; 2 Tuberculosis Elimination Program, Metro Public Health Department, Nashville, USA; 3 Tuberculosis Elimination Program, Tennessee Department of Health, Nashville, USA; 4 Department of Biostatistics, Vanderbilt University Medical Center, Nashville, USA; 5 Vanderbilt Tuberculosis Center, Vanderbilt University School of Medicine, Nashville, USA; 6 Division of Infectious Diseases, Department of Medicine, Vanderbilt University School of Medicine, A2200 Medical Center North, 1161 21st Avenue South, Nashville, TN 37232 USA; 7 The Aurum Institute, Johannesburg, South Africa

**Keywords:** Tuberculosis infection, Latent tuberculosis, Refugees, Tuberculosis prevention

## Abstract

**Background:**

Treatment of tuberculosis infection (TBI) in individuals at high risk for tuberculosis (TB) disease is a priority for TB elimination in the US. Newly arrived refugees in Middle Tennessee are screened for TBI, but factors associated with gaps in the TBI care cascade are not well characterized.

**Methods:**

We assessed the TBI care cascade from US entry to completion of treatment for refugees who resettled in Middle Tennessee from 2012 through 2016. We assessed factors associated with treatment initiation and completion using logistic regression models.

**Results:**

Of 6776 refugees who completed initial health screening, 1681 (25%) screened positive for TBI, 1208 were eligible for treatment, 690 started treatment, and 432 completed treatment. Male sex (Odds Ratio [OR]: 1.42; 95% Confidence Interval [CI]: 1.06, 1.89) and screening with interferon gamma release assay compared to tuberculin skin test (OR: 2.89; 95% CI: 1.59, 5.27) were associated with increased treatment initiation; living farther away from TB clinic was associated with decreased treatment initiation (OR: 0.91; 95% CI: 0.83, 0.99). Existing diabetes (OR: 7.27; 95% CI: 1.93, 27.30), receipt of influenza vaccination (OR: 1.65; 95% CI: 1.14, 2.40) and region of origin from South-Eastern or Southern Asia (OR_SEAsia_: 2.30; 95% CI: 1.43, 3.70; OR_SAsia_: 1.64; 95% CI: 1.02, 2.64) were associated with increased treatment completion. Six refugees developed TB disease after declining (*n* = 4) or partially completing (*n* = 2) TBI treatment; none who completed treatment developed TB disease.

**Conclusions:**

We determined gaps in the TBI care cascade among refugees in Middle Tennessee. Further assessment of barriers to treatment initiation and completion and interventions to assist refugees are warranted to improve these gaps and prevent TB disease.

## Background

Tuberculosis (TB) incidence rates in the US have been declining for several years, reaching a record low of 2.8 new cases per 100,000 persons in 2018 [1]. Foreign-born persons in the US accounted for 70% of reported TB cases in 2018. Molecular genotyping of *Mycobacterium tuberculosis* identified in US immigrant populations has shown that incident cases tend to be caused by reactivation or primary progression of TB infection (TBI) that occurs prior to US entry rather than local, US transmission of TB disease [2, 3].

In countries with low TB endemicity such as the US, identifying and treating TBI in individuals at high risk of TB (such as immigrants and refugees from TB endemic regions) is a priority for achieving TB elimination [4]. Despite comprehensive screening protocols [4, 5], TBI treatment initiation among foreign-born persons remains a challenge. Studies of immigrant populations have shown TBI treatment initiation rates ranging from 23% in North Carolina to 82% in Baltimore and Tennessee [6].

Refugees, defined as people who flee their country and are unwilling or unable to return due to a well-founded fear, can be resettled to the US after screening by multiple US security agencies while located outside of the US [7]. They represent a subset of the foreign-born population who are thoroughly vetted before arrival in the US [8] and for whom medical assessment data are systematically collected [9–11].

In this study, we sought to describe the TBI cascade of care for refugees in Middle Tennessee and assess factors associated with TBI treatment initiation and completion to inform targeted strategies to optimize TBI treatment initiation and completion rates.

Several factors are associated with failure to initiate and/or complete TBI treatment including the length of treatment, comorbidities, concern about side effects, and place of birth [12–17]. These associations are often inconsistent across studies and may vary regionally [18]. We sought to identify factors associated with gaps in the TBI treatment cascade among refugees in Tennessee.

## Methods

### Study design and population

We conducted a retrospective cohort study of refugees resettled in Middle Tennessee between January 1, 2012, and December 31, 2016, to characterize the TBI care cascade from 1) Initial TBI screening, 2) Follow-up evaluation for TBI positives, 3) Offer of TBI treatment, 4) Treatment initiation, to 5) Treatment completion. During the study period, all refugees relocated to Middle Tennessee were scheduled for an initial US health assessment at Siloam Family Health Center (Siloam) in Nashville, Tennessee (Supplementary Fig. 1). To assess factors associated with treatment initiation, we analyzed a subset of refugees who screened positive for TBI during their initial health assessment using a tuberculin skin test (TST) or interferon gamma release assay (IGRA) and were age 5 years or older at the time of resettlement. A positive screen was defined as a TST reaction of ≥10 mm of induration (≥5 mm if HIV positive) or a positive QuantiFERON (QFT) test [19]. Refugees were excluded from the analysis dataset if they 1) had TB disease at the time of evaluation at Metro Public Health Department (MPHD) in Nashville, TN; 2) had previously completed TBI treatment; 3) lived outside Davidson County; 4) were determined to have a false-positive screening test (i.e., a negative result on a follow-up TST or IGRA test); or 5) were advised by a provider not to start treatment. To assess factors associated with treatment completion, we excluded any refugees who did not start TBI treatment. Siloam and the Institutional Review Boards of Vanderbilt University and MPHD approved this study.

### Data sources and measurements

We used data from the Electronic Disease Notification System (EDN), a centralized electronic disease surveillance system used by the US Centers for Disease Control and Prevention (CDC) to identify all refugees who arrived in Middle Tennessee within the study period [20]. To identify refugees who screened positive for TBI, we used data from Siloam collected on the Tennessee Initial Refugee Health Assessment (TIRHA) form during initial screening visits. To determine treatment initiation, we abstracted data from the TB Risk Assessment Tool and the Patient Tracking Billing Management Information System (PTBMIS) at MPHD. To determine treatment completion, we abstracted TBI medication dosing data from PTBMIS at MPHD and used data from medical record review and the National Notifiable Disease Surveillance System (NNDSS) Base System (NBS), a data information system used to manage reportable disease data in Tennessee [21]. Because there was no unique identifier across databases, we used approximate string-matching techniques to match patients (Supplementary Tables 1A and 1B).

Eligible persons were treated for TBI with one of three regimens according to patient and provider decision: 1) 9 months of once-daily isoniazid (9H); 2) 4 months of once-daily rifampin (4R); or 3) starting in fall 2015, 3 months of observed once-weekly isoniazid and rifapentine (3HP). We defined treatment initiation as observed ingestion of the first dose of 3HP, or patient pick up of the first month’s supply of medication of 9H or 4R. We assessed treatment details using procedure codes in PTBMIS and by manual medical record review for details unable to be determined in PTBMIS. We assessed whether individuals met CDC guidelines for starting treatment within 60 days of screening positive for TBI and each individual’s treatment completion status 1 year (365 days) after the treatment start date. Treatment completion was defined as 1) having a “treatment complete” procedure code in the medical record, or 2) documentation of “treatment complete” by a healthcare professional in the medical record within 1 year from the start date.

We evaluated comorbidities in two ways. First, we estimated the overall comorbidity burden using the Elixhauser Comorbidity Index [22]. We assigned Elixhauser scores based on screening test results and ICD-9-CM codes recorded during the initial screening visit [23]. The study population was dichotomized into two groups: Elixhauser Score = 0 (No Comorbidity) or Elixhauser Score ≥ 1 (Any Comorbidity). Second, we evaluated four individual comorbidities including HIV, diabetes, liver disease and hypertension where the first three were associated with increased risk of TB disease [19, 24, 25] and the last one was the most commonly occurring comorbidity in the population. We also assessed age, sex, smoking history (any tobacco use: yes or no), alcohol abuse (excessive drinking of alcohol as determined by screening physician), type of screening test (TST or IGRA), distance travelled to TB clinic (straight line distance from home address to clinic), region of origin (created by categorizing countries of origin into regions based on the UN Standard Country or Area Codes for Statistical Use [26]), region of routing (created by categorizing countries where overseas medical exams were performed [26]), flu shot received at initial health assessment (yes or no), days to screening appointment from date of arrival in the US, year of arrival in the US, family size at time of entry (single, 2–3 persons, or > 3 individuals), and regimen (9H, 3HP, 4R or a combination). Geo-coded home addresses were used to calculate the straight line distance from home to the TB clinic by BatchGeo [27] to visualize the distribution and density for the study population.

We matched cases of TB disease from PTBMIS and NBS to the list of refugees screened for TBI at Siloam to determine whether any of them developed TB disease. We reviewed the medical record of all cases of TB disease that matched our screening dataset.

### Statistical analysis

We described variables of interest using proportions for categorical variables and medians and interquartile ranges for continuous variables. We compared demographic and clinical characteristics by comorbidity burden using chi-squared tests for categorical variables and Wilcoxon rank-sum tests for continuous variables. We conducted univariate and multiple logistic regression models to evaluate potential risk factors associated with TBI treatment initiation and completion. Predetermined factors and those with *P* < 0.1 in univariate analyses were used in the multiple logistic regression model, including age, sex, screening test type, year of arrival, family size at time of entry, and distance to the TB clinic. Models using either comorbidity burden or individual comorbidities (HIV, diabetes, liver disease, hypertension) were performed separately. To model treatment completion, we included the covariates used in the treatment initiation model along with receipt of influenza vaccination, region of origin and days from arrival to screening appointment with or without interaction terms between sex and family size and between sex and region of origin. The likelihood ratio test was used to compare these two treatment completion models. Since no significant differences were found, results of the model without interaction terms were reported. We also applied restricted cubic splines on days from arrival to screening appointment with three knots due to the concern of skewed data distribution.

All data analyses were conducted using SAS Studio 3.6 (SAS Institute Inc., Cary, NC) and Stata 14.2 (Stata Corporation, College Station, TX, USA).

## Results

### Participant characteristics

**Fig. 1:**
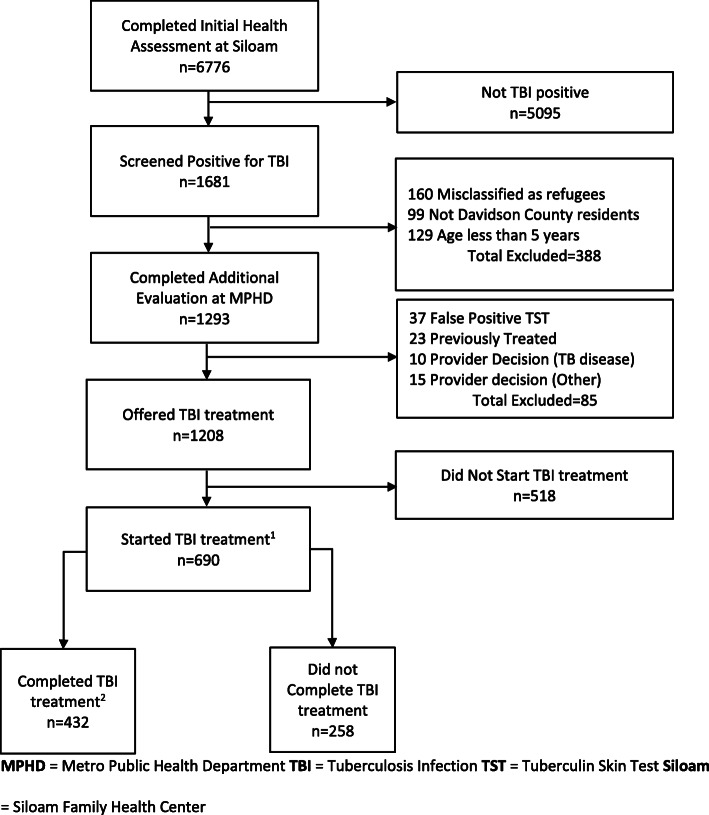
Flow Diagram of Individuals Included in Analysis of TBI Treatment Initiation and Completion. ^1^Of these, 294 individuals started treatment within 60 days of screening positive and were included in the treatment initiation regression analyses. ^2^Of these, 281 completed treatment within 365 days of starting treatment and were included in the treatment completion regression analyses

Of 6776 individuals who completed an initial health assessment in the 5-year study period, 1681 (25%) screened positive for TBI. Of these, we excluded 388 individuals from the analysis, and at the MPHD an additional 85 refugees were found to be ineligible for TBI treatment (Fig. 1).

The study population of 1208 refugees offered TBI treatment was made up predominantly of African refugees from Somalia, D.R. Congo, and Sudan as well as Asian refugees from Burma, Bhutan, and Iraq. Nepali, Somali and Arabic were the most commonly spoken languages and less than 3% reported English as their primary language. The median age was 31 years. About 34% of the study population were in the Any Comorbidity Group with the most common comorbidities being uncomplicated hypertension, obesity, liver disease, and uncomplicated diabetes mellitus (Supplementary Table 2). Individuals in the Any Comorbidity Group were significantly older than those in the No Comorbidity Group, were more likely to be smokers, to not have received influenza vaccination, and to have entered the country in a family of 2 or 3 (Table 1).

**Table 1:** Population Demographics and Selected Characteristics by Comorbidity Index

Demographic	Elixhauser Score = 0 (No Comorbidity)	Elixhauser Score ≥ 1 (Any Comorbodity)
Sex		
Male, *[n,%]*	478 (60)	238 (58)
Age		
Years [*median, IQR*]	28 (21–36)	39 (30–51)
Distance to TB clinic		
Miles [*median, IQR*]	8.3 (7.7–9.3)	8.4 (7.9–9.3)
Time to Screening Appointment		
Days [*median, IQR*]	27 (19–34)	26 (19–34)
Region of Origin		
*Northern Africa, [n,%]*	29 (4)	16 (4)
*Sub-Saharan Africa, [n,%]*	264 (33)	128 (31)
*Central Asia, [n,%]*	1 (< 1)	0 (0)
*South-Eastern Asia, [n,%]*	225 (28)	99 (24)
*Southern Asia, [n,%]*	223 (28)	98 (24)
*Western Asia, [n,%]*	52 (7)	65 (16)
*Latin America & Caribbean, [n,%]*	3 (< 1)	5 (1)
Region of Routing		
*Northern Africa, [n,%]*	25 (3)	12 (3)
*Sub-Saharan Africa, [n,%]*	194 (24)	96 (23)
*Central Asia, [n,%]*	1 (< 1)	0 (0)
*Eastern Asia, [n,%]*	2 (< 1)	0 (0)
*South-Eastern Asia, [n,%]*	192 (24)	90 (22)
*Southern Asia, [n,%]*	202 (25)	85 (21)
*Western Asia, [n,%]*	56 (7)	63 (15)
*Latin America & Caribbean, [n,%]*	2 (< 1)	5 (1)
*Eastern Europe, [n,%]*	0 (0)	1 (< 1)
*Northern Europe, [n,%]*	1 (< 1)	0(0)
*Southern Europe, [n,%]*	21 (3)	6(2)
Year of Arrival		
2012, *[n,%]*	250 (31)	102 (25)
2013, *[n,%]*	141 (18)	77 (19)
2014, *[n,%]*	137 (17)	80 (19)
2015, *[n,%]*	134 (17)	69 (17)
2016, *[n,%]*	135 (17)	83 (21)
Screening Test		
Tuberculin Skin Test, *[n,%]*	191 (24)	90 (22)
Interferon Gamma Release Assay, *[n,%]*	606 (76)	321 (78)
Smoking History		
Yes, *[n,%]*	37 (5)	40 (10)
Flu Shot Received		
Yes, *[n,%]*	450 (57)	201 (49)
Family Size at Time of Entry		
Single, *[n,%]*	369 (46)	177 (43)
Family of 2–3, *[n,%]*	165 (21)	115 (28)
Family of > 3, *[n,%]*	263 (33)	119 (29)
Primary Language		
English, *[n,%]*	3 (< 1)	3 (< 1)
Nepali, *[n,%]*	201 (25)	85 (21)
Somali, *[n,%]*	162 (20)	64 (16)
Arabic, *[n,%]*	64 (8)	59 (14)
Burmese, *[n,%]*	78 (10)	28 (7)
Swahili, *[n,%]*	53 (7)	25 (6)
Other, *[n,%]*	236 (30)	147 (36)
Pregnant		
Yes, *[n,%]*	17 (5)	8 (5)
**Total**	***N*** **= 797**	***N*** **= 411**

*IQR* Interquartile Range

### TBI treatment initiation

**Fig. 2:**
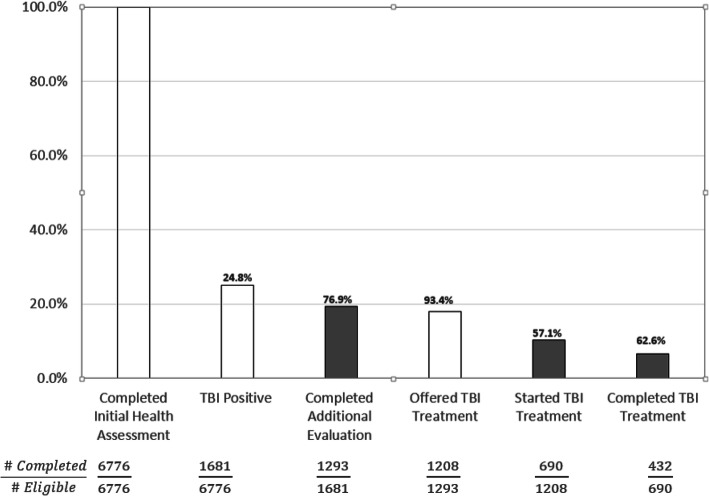
Tuberculosis Infection Treatment Cascade of Care. TBI care cascade among refugees resettled in Middle Tennessee from Jan 2012 to Dec 2016. The total numbers of individuals who completed each step as a proportion of those eligible to complete the step are given. Proportions (%) were calculated using the previous bar as the denominator for each step. Gray bars represent key gaps in the cascade related to screening completion, treatment initiation, and treatment completion. White bars represent gaps related to legitimate exclusions, i.e., individuals who had negative screening tests, lived outside Davidson County, had false positive test results, had previously completed TBI treatment, or were advised by a provider not to start treatment

Of the 1208 refugees eligible for treatment, 690 (54%) started treatment (Fig. 2). Of these, 294 (43%) started TBI treatment within 60 days of screening positive. The most common recorded reason for non-initiation of TBI treatment was declination of treatment (55%). Other reasons included loss to follow-up (8%) and relocation (5%) (Table 2).

**Table 2:** Reasons for Non-Initiation/ Non-Completion of TBI Treatment

Reason	Frequency
**Non-Initiation**	
*Declined Medication*	286 (55.2%)
*Lost to Follow up*	43 (8.3%)
*Moved Away*	28 (5.4%)
*Other*	7 (1.4%)
*Unknown*	154 (29.7%)
***Total***	**518**
**Non-Completion**	
*Lost to Follow up*	66 (25.6%)
*Declined Medication/ Chose to Stop*	43 (16.7%)
*Moved Away*	28 (10.9%)
*Provider Decision*	27 (10.5%)
*Adverse Effect of Medication*	6 (2.3%)
*Active TB Developed*	2 (0.8%)
*No TB found*	2 (0.8%)
*Previously Treated for TB/TBI*	1 (0.4%)
*Death*	1 (0.4%)
*Unknown*	82 (31.8%)
***Total***	**258**

In the univariate analysis, male sex, having an IGRA screening test rather than a TST and having an initial health assessment in 2013 were significantly associated with increased treatment initiation. Older age, living further away from the TB clinic and having entered the country in a family of 2 or 3 were significantly associated with decreased treatment initiation (Table 3). We did not find a significant association between Any Comorbidity and No Comorbidity groups, but when we assessed individual comorbidities, hypertension was associated with treatment initiation.

**Table 3:** Univariate and Multivariable Analysis of Factors Associated with TBI Treatment Initiation

	Univariate			Multivariable		
	OR	95% CI	p	OR	95% CI	p
Elixhauser Comorbidity Index^a^						
*No Comorbidity (Score = 0)*	*ref*					
*Any Comorbidity (Score ≥ 1)*	0.79	[0.60,1.05]	0.11			
Diabetes^a^						
*No*	*ref*					
*Yes*	0.68	[0.31,1.48]	0.34			
Hypertension^a^						
*No*	*ref*					
*Yes*	0.54	[0.33,0.88]	0.01	0.64	[0.37,1.10]	0.10
Liver Disease^a^						
*No*	*ref*					
*Yes*	0.94	[0.54,1.65]	0.83			
HIV^a^						
*No*	*ref*					
*Yes*	1.17	[0.31,4.43]	0.82			
Screening Test						
*Tuberculin Skin Test*	*ref*					
*Interferon Gamma Release Assay*	1.88	[1.33,2.67]	< 0.001	2.89	[1.59,5.27]	0.001
Year of Arrival						
*2012*	*ref*					
*2013*	2.17	[1.49,3.18]	< 0.001	1.14	[0.66,1.99]	0.64
*2014*	1.42	[0.96,2.12]	0.08	0.72	[0.39,1.33]	0.29
*2015*	1.41	[0.94,2.12]	0.09	0.66	[0.36,1.21]	0.18
*2016*	0.73	[0.47,1.14]	0.17	0.40	[0.21,0.74]	0.004
Family Size at Time of Entry						
*Single*	*ref*					
*Family of 2–3*	0.82	[0.58,1.14]	0.23	1.04	[0.71,1.52]	0.83
*Family of > 3*	0.73	[0.53,0.99]	0.04	1.03	[0.72,1.48]	0.87
Sex						
*Female*	*ref*					
*Male*	1.44	[1.09,1.89]	0.01	1.42	[1.06,1.89]	0.02
Region of Origin						
*Sub-Saharan Africa*	*ref*			*ref*		
*South-eastern Asia*	*1.18*	[0.85,1.66]	0.32	1.07	[0.75,1.54]	0.69
*Southern Asia*	*0.97*	[0.69,1.37]	0.86	1.05	[0.71,1.54]	0.82
*Western Asia*	*0.76*	[0.46,1.28]	0.30	0.98	[0.57,1.69]	0.93
*Other*	0.80	[0.40,1.61]	0.53	0.87	[0.42,1.78]	0.70
Smoking History						
*No*	*ref*					
*Yes*	0.8	[0.45,1.41]	0.44			
Flu Shot Received						
*No*	*ref*					
*Yes*	0.95	[0.73,1.24]	0.73			
Distance to TB Clinic, miles	0.92	[0.85,1.00]	0.04	0.91	[0.83,0.99]	0.04
Time to Appointment, days						
*Screened after 11* vs *28 days*	0.96	[0.55,1.68]	0.90	1.01	[0.63,1.60]	0.98
*Screened after 14* vs *28 days*	0.96	[0.70,1.27]	0.71	1.02	[0.74,1.40]	0.91
Age^b^	0.99	[0.98,1.00]	0.04	0.99	[0.98,1.00]	0.28

^a^Elixhauser Comorbidity Score and individual comorbidities were run in separate models

^b^Odds expressed as change per one year increase in age

In the adjusted model, male sex and an IGRA screening test were significantly associated with increased odds of treatment initiation (Adjusted Odds Ratio [aOR] _Male_: 1.44; 95% Confidence Interval [CI]: 1.08, 1.92; aOR_IGRA_: 2.79; 95% CI: 1.57, 4.93). Living farther away from the TB clinic was significantly associated with decreased odds of treatment initiation (aOR: 0.91; 95% CI: 0.83, 0.99). A higher percentage of non-initiators (50%) lived more than the median straight-line distance from the TB clinic than initiators (47%), however this difference was not statistically significant. We did not find a significant interaction between sex and family size or sex and region of origin.

### TBI treatment completion

Of the 690 refugees who started treatment, 432 (63%) completed treatment; 281 (41%) did so within a year of treatment initiation. Non-completion was due to loss to follow-up, declination of further treatment, relocation, and provider decisions to terminate treatment early (Table 3). In the univariate analysis, receipt of influenza vaccination during the screening appointment and having a region of origin in Southern or South-eastern Asia were significantly associated with increased treatment completion (Table 4). We did not find a significant association between Any Comorbidity and No Comorbidity groups, but when we assessed individual comorbidities, diabetes was associated with increased treatment completion. Additionally, we found a statistically significant nonlinear relationship between the duration from US arrival to screening appointment and treatment completion.

**Table 4:** Univariate and Multivariable Analysis of Factors Associated with TBI Treatment Completion

	Univariate			Multivariable		
	OR	95% CI	p	OR	95% CI	p
Elixhauser Comorbidity Index^a^						
*No Comorbidity (Score = 0)*	*ref*					
*Any Comorbidity (Score ≥ 1)*	1.10	[0.77,1.56]	0.61			
Diabetes^a^						
*No*	*ref*			*ref*		
*Yes*	5.46	[1.57,18.94]	0.008	7.27	[1.93,27.30]	0.003
Hypertension^a^						
*No*	*ref*					
*Yes*	1.29	[0.84,1.19]	0.37			
Liver Disease^a^						
*No*	*ref*					
*Yes*	1.05	[0.50,2.21]	0.90			
HIV^a^						
*No*	*ref*					
*Yes*	0.73	[0.16,3.28]	0.68			
Screening Test						
*Tuberculin Skin Test*	*ref*					
*Interferon Gamma Release Assay*	1.21	[0.80,1.84]	0.37			
Year of Arrival						
*2012*	*ref*					
*2013*	1.50	[0.93,2.41]	0.10			
*2014*	1.32	[0.79,2.18]	0.29			
*2015*	1.12	[0.66,1.89]	0.67			
*2016*	0.99	[0.58,1.70]	0.97			
Family Size at Time of Entry						
*Single*	*ref*			*ref*		
*Family of 2–3*	1.36	[0.89,2.07]	0.16	1.18	[0.74,1.87]	0.49
*Family of > 3*	1.05	[0.71,1.56]	0.80	1.11	[0.70,1.74]	0.66
Sex						
*Female*	*ref*			*ref*		
*Male*	1.01	[0.72,1.43]	0.06	1.02	[0.74,1.49]	0.90
Region of Origin						
*Sub-Saharan Africa*	*ref*			*ref*		
*South-eastern Asia*	2.10	[1.34,3.29]	0.001	2.30	[1.43,3.70]	0.001
*Southern Asia*	1.63	[1.05,2.58]	0.03	1.64	[1.02,2.64]	0.04
*Western Asia*	0.88	[0.44,1.77]	0.72	0.64	[0.29,1.38]	0.25
*Other*	2.00	[0.84,4.79]	0.12	2.33	[0.92,5.94]	0.08
Smoking History						
*No*	*ref*					
*Yes*	0.92	[0.47,1.79]	0.80			
Flu Shot Received						
*No*	*ref*			*ref*		
*Yes*	1.51	[1.08,2.12]	0.02	1.65	[1.14,2.40]	0.008
Regimen						
*9H: Isoniazid only*	*ref*			*ref*		
*3HP: Isoniazid + Rifapentine*	1.33	[.087,2.04]	0.19	1.58	[0.99,2.51]	0.05
*4R: Rifampin*	1.49	[0.47,4.79]	0.50	1.47	[0.42,5.10]	0.54
*Multiple regimens*	1.65	[0.75,3.61]	0.21	1.54	[0.68,3.47]	0.30
Time to Appointment, days						
*Screened after 11* vs *28 days*	0.49	[0.28,0.86]	0.01	0.47	[0.26,0.86]	0.01
*Screened after 14* vs *28 days*	0.61	[0.41,0.91]	0.01	0.48	[0.27,0.86]	0.01
Distance to TB Clinic, miles	0.92	[0.82,1.03]	0.16			
Age^b^	1.00	[0.99,1.01]	0.79	1.00	[0.99,1.02]	0.63

^a^Elixhauser Comorbidity Score and individual comorbidities were run in separate models

^b^Odds expressed as change per one year increase in age

In the adjusted model, the associations remained significant (aOR _Diabetes_: 7.27; 95% CI: 1.93, 27.30; aOR _Flu_: 1.65; 95% CI: 1.14, 2.40; OR_SEAsia_: 2.30; 95% CI: 1.43, 3.70; OR_SAsia_: 1.64; 95% CI: 1.02, 2.64). We modeled the nonlinear association between days to screening appointment and treatment completion using restricted cubic splines (Supplementary Fig. 2). Adjusting for other covariates, the odds of completing treatment decreased for individuals who were screened earlier (e.g., 11 or 14 days after arrival into the US) compared to those who were screened later (28 days after arrival into the US) (aOR _Screen (11 vs 28)_: 0.47; 95% CI: 0.26, 0.86; aOR _Screen (14 vs 28)_: 0.48; 95% CI: 0.27, 0.86). Given this finding, we also included a non-linear term for days to screening appointment in the multivariable treatment initiation model, however this was not significant.

### TB disease

Of the 6776 individuals who were screened for TBI between 2012 and 2016, 21 (0.3%) developed TB disease during the study period (Supplementary Fig. 3). All 21 individuals who developed TB disease screened positive for TBI at their initial health visit Siloam. Of the 21 cases, 15 (71%) were diagnosed with TB disease at the follow-up clinical evaluation at MPHD, with a median time from US entry to TB disease diagnosis at MPHD (time to diagnosis) of 64 days. Of the remaining 6 cases, 4 individuals declined TBI treatment due to pregnancy (*n* = 3) or issues with transportation (*n* = 1) with a median time to diagnosis of 478 days. The other 2 cases were reported in individuals who had partially completed treatment on the 9H regimen (33 and 66% completed) with 602 days and 591 days to diagnosis respectively. One additional refugee developed TB disease 15 days after US entry and was referred directly to MPHD for treatment before having an initial health screen. There were no reported cases of TB disease among refugees who completed TBI treatment in our dataset.

## Discussion

In our study, we demonstrated that the largest gaps in the TBI cascade of care for refugees were TBI treatment initiation and treatment completion. Notably, the refugees diagnosed with TB disease during the study period were diagnosed after referral to MPHD from their initial health screening (suggesting imported TB disease), or among those who did not initiate or complete TBI treatment. None of the refugees who screened negative for TBI or completed TBI treatment developed active TB in the study period. Although persistent risk of TBI reactivation has been documented in immigrants, our findings suggest that treatment of TBI in this high-risk group may be effective [28].

Distance to TB clinic was notable among factors associated with TBI treatment initiation. For every mile individuals lived from the TB clinic, the odds of treatment initiation dropped by about 9 %. Individuals who lived further away from the TB clinic may have had difficulty with transportation, whether by private vehicle, availability of others to provide transportation, or access to public transportation. The travel distance and times may have been prohibitively time-consuming in relation to work or family obligations.

Reasons that male sex was associated with increased odds of treatment initiation are unclear. We did not find other studies reporting sex differences among refugee initiation of TBI treatment; one study of refugees in Canada did not find sex differences associated with completion of TBI treatment [29]. We considered the possibility that females may have had competing responsibilities, but we did not find a significant interaction between sex and family size [30]. Family size counts, however, did not include refugees in the same family who arrived in different years.

Refugees who were screened for TBI with an IGRA had over twice the odds of those who were screened with a TST to start TBI treatment. The delayed interpretation of TST likely did not contribute substantially to the difference since all refugees were scheduled for a follow-up visit at Siloam two to 3 days after the initial visit regardless of whether they received an IGRA or TST. Rather, individuals with positive TST results may have believed that they had false positive tests due to prior Bacille Calmette-Guerin (BCG) vaccination [31]. Additionally, since IGRAs eventually replaced TST at Siloam, improvements in referral to the TB clinic over time or provider confidence in IGRA tests may have contributed to the difference.

Individuals who received influenza vaccination had almost one and a half times increased odds of completing treatment compared to those who did not. The influenza vaccine is optional and offered free of charge during the initial health assessment at Siloam. Perceived risk is an important component of health-related decision making [32, 33], and individuals who do not perceive themselves to be at risk of developing a disease are less likely to receive medication or vaccinations [34, 35]. Opting to receive the vaccine may therefore be a proxy for adherence to other optional treatments such as TBI treatment. Individuals who completed their initial screening visit early after arrival into the country had a marked decrease in the odds of completing treatment when compared to those who screened twenty-eight days after arrival. The CDC guidelines recommend completing TBI screening and starting treatment within 90 days of US arrival [10]. Within this window, refugees must complete several resettlement procedures including finding employment, registering for social services, and enrolling children in school [36–38] some of which are tied to benefits such as refugee cash assistance. Juggling these priorities can be overwhelming particularly during the first weeks and individuals who are screened later, and therefore start treatment later, may have had more time to acclimate and complete other activities that could compete with completing treatment.

Many interventions to address the gaps we identified in the TBI care cascade are already in use at MPHD. These include: 1) using IGRA instead of TST to screen for TBI whenever possible, 2) provision of transport vouchers to assist with cost of travel to the TB clinic, 3) evening clinic hours on selected days to accommodate appointments outside of regular working hours, and 4) dedicated case managers who perform routine follow-up to ensure treatment completion. In addition to these, further interventions such as implementing electronic Directly Observed Therapy (eDOT) and providing alternate locations for medication pick-up closer to immigrant communities could help increase treatment initiation rates.

Our study has several limitations. First, data were linked using approximate matching techniques which could have caused outcome misclassification of individuals who sought treatment at other facilities or those who were not linked in the dataset. Thus, the proportions reported in our treatment cascade could be an underestimation of the true gaps. Second, we had some missing and incomplete data. For example, due to the nature of TBI screening of refugees in Middle Tennessee, only individuals determined to be at high risk were screened for hepatitis C [39] – one of the comorbidities included in our calculation of comorbidity burden. However, given the low prevalence of Hepatitis C in our study population (about 3%), this was likely not a major source of bias. Finally, this study is limited in its generalizability because of the unique make-up of the refugees in Middle Tennessee. We were able, however, to provide context-specific determinants of treatment initiation and completion to inform local practices and potential interventions.

## Conclusion

In this retrospective cohort study, we described gaps in the TBI care cascade among refugees in Middle Tennessee and identified factors associated with TBI treatment initiation and completion. Interventions (i.e., satellite clinics and video observation of medication administration) to assist refugees who live further away from the TB clinic or who may be juggling competing priorities may increase treatment initiation and completion rates.

## Supplementary information

**Additional file 1 Supplementary Table 1A.** Matching Rules and Algorithms for Identifying Participants across Study Datasets. **Supplementary Table 1B.** Breakdown of Participants by Matching Conditions. **Supplementary Table 2.** Distribution of Elixhauser Comorbidity Groups within Study Population (*n* = 1208). Individuals could have multiple comorbidities. **Supplementary Fig. 1.** TB infection screening workflow in Middle Tennessee. **Supplementary Fig. 2.** Association between days from US arrival to initial screening appointment and TBI treatment completion. Days to screening appointment is modeled as a continuous variable using restricted cubic splines with 3 knots. In the final logistic model, covariates are adjusted using the following levels: sex (female), Elixhauser comorbidity index (score < 1), receipt of influenza vaccine (yes), country of origin (not Asian); regimen (isoniazid only [9H]). **Supplementary Fig. 3.** Diagnosis of Tuberculosis Disease (*N* = 22) among Refugees in Middle Tennessee.

## Data Availability

The datasets generated and/or analyzed during the current study are not publicly available because they contain information that could compromise research participant privacy but are available from the corresponding author on reasonable request.

## Abbreviations

3HP: A three-month regimen of once-weekly isoniazid and rifapentine
4R: A four-month regimen of once-daily rifampin
9H: A nine-month regiment of once-daily isoniazid
BCG: Bacille Calmette-Guérin
CDC: Centers for Disease Control and Prevention
CTSA: Clinical and Translational Science Award
EDN: Electronic Disease Notification
eDOT: Electronic directly observed therapy
ICD: International Classification of Diseases
IGRA: Interferon gamma release assay
IQR: Interquartile range
MPHD: Metro Public Health Department
NBS: National Base System
NNDSS: National Notifiable Disease Surveillance System
PTBMIS: Patient Tracking and Billing Management Information System
QFT: QuantiFERON TB test
RAT: Risk assessment tool
SAT: Self-administered therapy
Siloam: Siloam Family Health Center
TB: Tuberculosis
TBI: Tuberculosis infection
TDH: Tennessee Department of Health
TIRHA: Tennessee Initial Refugee Health Assessment
TST: Tuberculin skin test
VOLAG: Volunteer agency
