# Supplementary material for: Gaps in the care cascade for screening and treatment of refugees with tuberculosis infection in Middle Tennessee: a retrospective cohort study
Source: BMC Infect Dis. 2020 Aug 10;20:592. doi: 10.1186/s12879-020-05311-0 (PMC7418421; doi:10.1186/s12879-020-05311-0)
Supplement: Supplementary file 1 — Additional file 1 Supplementary Table 1A. Matching Rules and Algorithms for Identifying Participants across Study Datasets. Supplementary Table 1B. Breakdown of Participants by Matching Conditions. Supplementary Table 2. Distribution of Elixhauser Comorbidity Groups within Study Population (n = 1208). Individuals could have multiple comorbidities. Supplementary Fig. 1. TB infection screening workflow in Middle Tennessee. Supplementary Fig. 2. Association between days from US arrival to initial screening appointment and TBI treatment completion. Days to screening appointment is modeled as a continuous variable using restricted cubic splines with 3 knots. In the final logistic model, covariates are adjusted using the following levels: sex (female), Elixhauser comorbidity index (score < 1), receipt of influenza vaccine (yes), country of origin (not Asian); regimen (isoniazid only [9H]). Supplementary Fig. 3. Diagnosis of Tuberculosis Disease (N = 22) among Refugees in Middle Tennessee. [file 12879_2020_5311_MOESM1_ESM.docx]

**Supplementary Tables and Figures**

Gaps in the Care Cascade for Screening and Treatment of Refugees with Tuberculosis Infection in Middle Tennessee: A Retrospective Cohort Study

**Supplementary Table 1A:** Matching Rules and Algorithms for Identifying Participants across Study Datasets

| **Matching Methods** | **Variables** | **Matching Algorithm** | **Command (Software)** | **Threshold** |
| --- | --- | --- | --- | --- |
| Exact | Full Name  Date of Birth  Sex | Exact |  |  |
| Fuzzy | Full Name  First Name  Last Name | Soundex  Generalized Edit Distance  Levenshtein Edit Distance  Last Name, First Initial  Inverted First and Last Name | SOUNDEX function (SAS) & Soundex() code (Stata)  COMPGED function (SAS)  COMPLEV function (SAS) & STRDIST module (Stata) | <750  <7 |
| Fuzzy | Date of Birth | DOB +/- 365 days  YOB +/- 10 years |  |  |

**Supplementary Table 1B:** Breakdown of Participants by Matching Conditions

| **Matching Conditions** | **Total** | **Cum Total** | **Cum %** |
| --- | --- | --- | --- |
| Exact Full Name, Exact Date of Birth, Exact Sex | 1111 | 1111 | 66.1 |
| Exact Full Name, Exact Sex, Fuzzy Date of Birth | 32 | 1143 | 67.9 |
| Exact Date of Birth, Exact Sex, Fuzzy Full Name | 128 | 1271 | 75.6 |
| Exact Date of Birth, Exact Sex, Fuzzy Last Name | 10 | 1281 | 76.2 |
| Exact Last Name, Exact First Initial, Exact Date of Birth, Exact Sex | 70 | 1351 | 80.3 |
| Exact Last Name, Exact First Initial, Exact Sex, Fuzzy Year of Birth | 9 | 1360 | 80.9 |
| Exact Date of Birth, Fuzzy Full Name | 17 | 1377 | 81.9 |
|  | **Overall Totals** | | |
| **Total who screened positive for tuberculosis** | **1681** | **1681** | **100.00** |
| **Matched to Metro Public Health Department visits** | **1377** | **1377** | **81.9** |
| **Included in analyses^1^** | **1293** | **1293** | **76.9** |

^1^84 individuals who matched at Metro Public Health Department were later excluded based on study inclusion/exclusion criteria

**Supplementary Table 2**: Distribution of Elixhauser Comorbidity Groups within Study Population (n=1208). Individuals could have multiple comorbidities.

| Elixhauser Comorbidity Group | Freq | % |
| --- | --- | --- |
| *None* | 797 | 65.98 |
| *Hypertension, Uncomplicated* | 151 | 12.50 |
| *Obesity* | 150 | 12.42 |
| *Liver Disease* | 77 | 6.37 |
| *Diabetes, Uncomplicated* | 47 | 3.89 |
| *Depression* | 35 | 2.90 |
| *Weight Loss* | 22 | 1.82 |
| *Chronic Pulmonary Disease* | 20 | 1.66 |
| *Coagulopathy* | 19 | 1.57 |
| *Hypothyroidism* | 17 | 1.41 |
| *Alcohol Abuse* | 15 | 1.24 |
| *HIV/AIDS* | 14 | 1.16 |
| *Cardiac Arrhythmias* | 10 | 0.83 |
| *Fluid and Electrolyte Disorders* | 5 | 0.41 |
| *Valvular Disease* | 5 | 0.41 |
| *Other Neurological Disorders* | 4 | 0.33 |
| *Peptic Ulcer Disease Excluding Bleeding* | 3 | 0.25 |
| *Psychoses* | 3 | 0.25 |
| *Renal Disease* | 3 | 0.25 |
| *Rheumatoid Arthritis/ Collagen Vascular* | 3 | 0.25 |
| *Paralysis* | 2 | 0.17 |
| *Pulmonary Circulation Disorders* | 2 | 0.17 |
| *Congestive Heart Failure* | 1 | 0.08 |
| *Metastatic Cancer* | 1 | 0.08 |
| *Peripheral Vascular Disorders* | 1 | 0.08 |
| *Solid Tumour without Metastasis* | 1 | 0.08 |
| *Blood Loss Anaemia* | 0 | 0.00 |
| *Deficiency Anaemia* | 0 | 0.00 |
| *Diabetes, Complicated* | 0 | 0.00 |
| *Drug Abuse* | 0 | 0.00 |
| *Hypertension, Complicated* | 0 | 0.00 |
| *Lymphoma* | 0 | 0.00 |

**
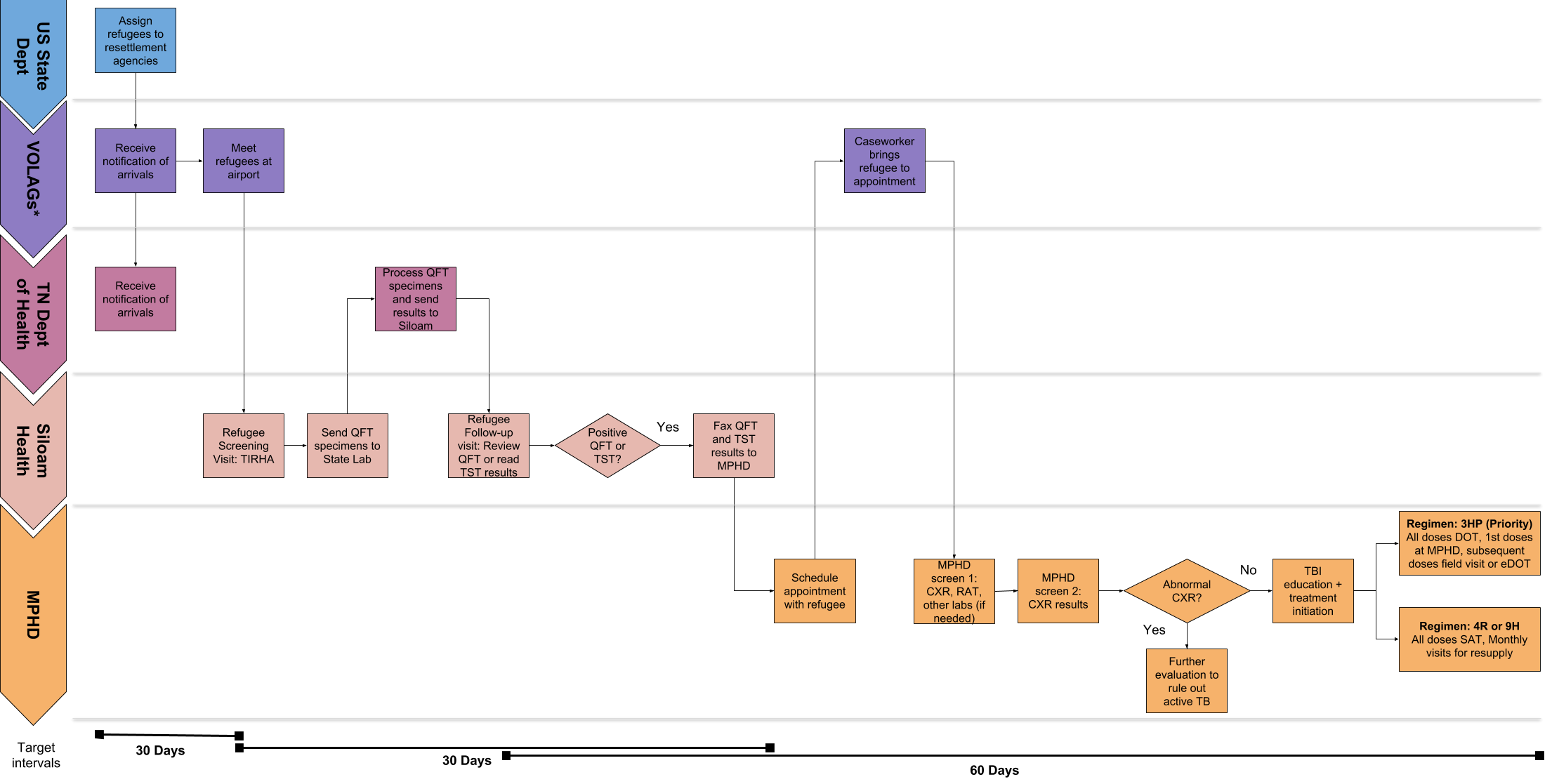
**

**Supplementary Figure 1:** TB infection screening workflow in Middle Tennessee.

**VOLAG** = Volunteer Agency; **TN Dept of Health** = Tennessee Department of Health; **MPHD** = Metro Public Health Department; **QFT** = QuantiFERON TB test; **TIRHA** = Tennessee Initial Refugee Health Assessment; **TST** = Tuberculin Skin Test; **CXR** = Chest X-ray; **RAT** = TB Risk Assessment Tool; **DOT** = Directly Observed Therapy; **SAT** = Self-Administered Therapy; **3HP** = 12 week, once-weekly isoniazid and rifapentine regimen; **4R** = 4 month, once-daily rifampin regimen; **9H** = 9 month, once-daily isoniazid regimen

**
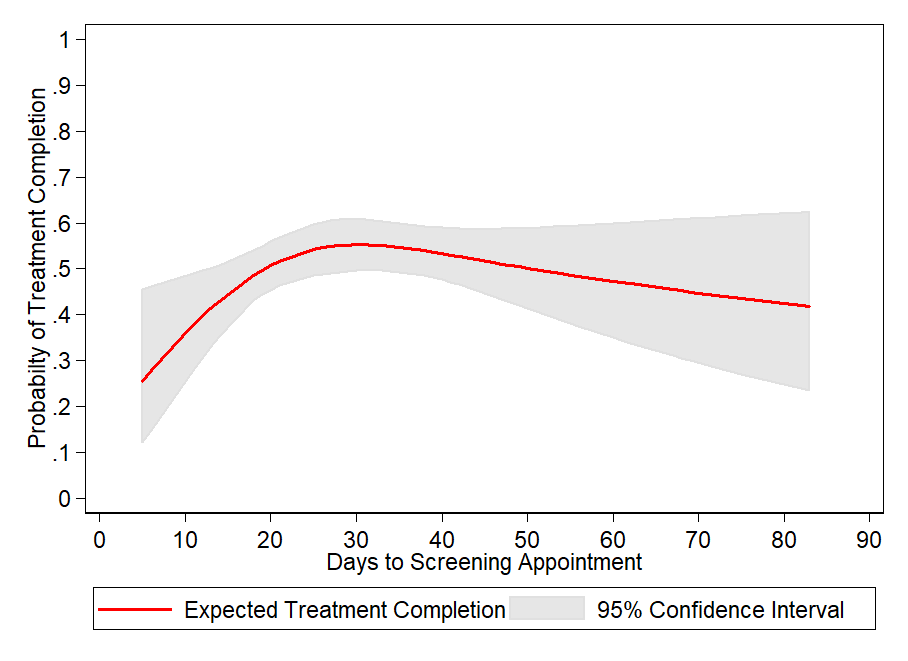
**

**Supplementary Figure 2:** Association between days from US arrival to initial screening appointment and TBI treatment completion. Days to screening appointment is modeled as a continuous variable using restricted cubic splines with 3 knots. In the final logistic model, covariates are adjusted using the following levels: sex (female), Elixhauser comorbidity index (score < 1), receipt of influenza vaccine (yes), country of origin (not Asian); regimen (isoniazid only [9H]).

**
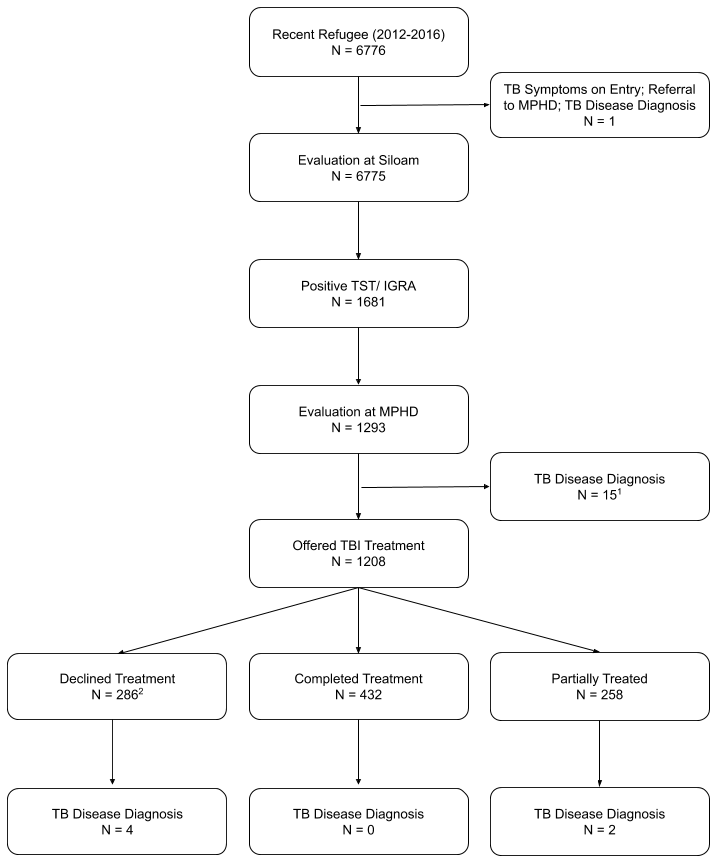
**

**Supplementary Figure 3:** Diagnosis of Tuberculosis Disease (N=22) among Refugees in Middle Tennessee

**MPHD =** Metro Public Health Department**; TST =** Tuberculin Skin Test**; IGRA =** Interferon Gamma Release Assay**; TBI =** Tuberculosis Infection

^1^ In addition to the 15 individuals diagnosed with TB disease, 70 others were ineligible to start treatment due to a false positive test, prior treatment or provider decision

^2^ In addition to the 286 individuals who declined treatment, 232 others did not start treatment for other reasons such as loss to follow-up and patient relocation.
